# Comment to: analysing topics using different methods promotes constructive debates. Author's reply

**DOI:** 10.1007/s10029-020-02205-7

**Published:** 2020-05-18

**Authors:** U. Klinge

**Affiliations:** Department of General-, Visceral- and Transplant Surgery, University Hospital of the RWTH Aachen, Pauwelsstraße 30, 52070 Aachen, Germany

In November 2019 Gavriilidis et al. compared total extraperitoneal endoscopic hernioplasty (TEP) with Lichtenstein hernioplasty by updated traditional and cumulative meta-analysis of randomised controlled trials (RCT) [1]. They concluded that the data revealed significantly higher rates of recurrence and vascular injuries in the TEP cohort compared to the Lichtenstein cohort.

In a letter to the editor Köckerling and Adolf pointed out that without the two largest studies (from 2004 and 2008) the remaining 12 of 14 RCTs in contrast showed a higher overall recurrence rate for the Lichtenstein operation. These two dominated the results of the meta-analysis because of the large sample size and number of recurrences [2].

Furthermore, in January 2020 Rosenberg and Andresen wrote that Gavriilidis et al. came to the wrong conclusion, not least because of the marked heterogeneity of the studies included, and that a conclusion based on the available data should be that there is no difference in the recurrence rates between the TEP and Lichtenstein repair [3].

In their subsequent reply Gavrilidis et al. admitted that their study was indeed inconclusive, and propose multicentre RCTs with predefined outcome estimation, size of hernial defects and size of mesh used, plus evaluation of cost effectiveness and the experience of the operator(s) to determine the possible superiority of one method over the other [4].

So, the solution to the question, which of the two procedures is better, once again is postponed to future, bigger RCTs. Even this may not be enough; it is a difficult problem to declare one procedure superior to another:

1. The studies used for the meta-analysis of Gavriilidis et al. include several without any complications. Obviously, in some hands TEP (*n* = 4 studies) as well as Lichtenstein (*n* = 3) can be performed without any recurrence or pain. Recurrence and pain therefore do not represent inherent and unavoidable risks. For some experts any attempt to define a “better” technique will be pointless, as both techniques can be regarded as “best”.
2. The focus on recurrence as the main for quality of a procedure is based on the assumption that all recurrences are caused by an inherent problem of the procedure, which certainly is not the case. Development of a recurrence may reflect the patients’ biology or immunology, the anatomy, a sufficient mesh overlap, and/or the experience of the surgeon, accordingly. Only a percentage of recurrence is strictly related to the procedure—and might have been avoided if another procedure had been used. Probably only 20% of recurrence cannot be related to individual risks, and therefore can be used as benchmark for the quality of the procedure. The procedure can therefore be responsible for only a small fraction of the recurrence rate. Of the 100 patients who made the difference between 4 and 6% observed for Lichtenstein and TEP, how many are free of extra-procedural risks? Is it justified to condemn a procedure been successfully performed in thousands of patients because of this small difference? And generally speaking, what level of difference justifies abandoning such a procedure?
3. RCT’s and meta-analysis of RCT’s used for the comparison of procedures include widely standardized cohorts of patients, by strict exclusion criteria and randomization. Maybe 15% of the cohort is smoking, has a mean age of 50, and 5% suffer from severe co-morbidities. But there is no patient, who is smoking at 15%, or has 5% type II diabetes; they smoke or not, have diabetes or not. Though a mean risk profile of the standardized cohort can be calculated, this does not reflect the individual risks e.g. smoking, immunosuppression, et cetera.
4. There may be some subgroups of patients who benefit from a TEP and others from a Lichtenstein. For most of the patients there will be no different outcome, regardless which technique is used. The main question thus should not be which technique is superior in general, but in which patients TEP or Lichtenstein is better. Is there a subgroup of patients, e.g. maybe with a large direct hernia who need larger mesh overlap who can be treated favourably by TEP, or of patients with small indirect hernia but numerous co-morbidities who can be easily repaired by Lichtenstein?
5. Unfortunately RCT’s commonly do not allow analysis of subgroups with sufficient power. The use of 20 variables, which are suspected to interfere with the risk for recurrence, already result in more than 1 million subgroups. This is the miserium: many variables have more than yes/no, and do not relate to recurrence in a linear way, which further expands the number of possible subgroups. As more patients will lead to a power-law increase of variation and subgroups, even larger cohorts cannot overcome this *Curse of dimensionality* [5].
6. It is an illusion to expect even from very large RCT’s to test the risks in subgroups with sufficient statistical power.

RCT’s and meta-analysis are a valuable tool to compare the effect of an intervention in highly standardized cohorts. However, in consideration of the many confounders interfering with any read-out our surgical hernia patients often do not represent “standard” patients, and despite widely standardized procedures the individual treatment, therefore, will not end up in standard results to be comparable by RCT. At best some patients form subgroups with similar risks, who can be treated in a similar way with a similar outcome. We should focus on identifying these subgroups instead of looking for the general assessment of surgical procedures in abstract cohorts. In regard to the 20 million hernia repairs each year the alternative will not be to treat all patients by one best technique, either Lichtenstein or TEP, but to stratify the selection of the various procedures according to patients at risk.
